# Development of a standards‐based phenotype model for gross motor function to support learning health systems in pediatric rehabilitation

**DOI:** 10.1002/lrh2.10266

**Published:** 2021-05-05

**Authors:** Nikolas Koscielniak, Gretchen Piatt, Charles Friedman, Alexandra Vinson, Rachel Richesson, Carole Tucker

**Affiliations:** ^1^ Clinical and Translational Science Institute Wake Forest University School of Medicine Winston‐Salem North Carolina USA; ^2^ Department of Learning Health Sciences University of Michigan Medical School Ann Arbor Michigan USA; ^3^ Department of Health and Rehabilitation Sciences Temple University Philadelphia Pennsylvania USA

**Keywords:** learning health systems, pediatric rehabilitation, phenotypes, infrastructure

## Abstract

**Introduction:**

Research and continuous quality improvement in pediatric rehabilitation settings require standardized data and a systematic approach to use these data.

**Methods:**

We systematically examined pediatric data concepts from a pediatric learning network to determine capacity for capturing gross motor function (GMF) for children with Cerebral Palsy (CP) as a demonstration for enabling infrastructure for research and quality improvement activities of an LHS. We used an iterative approach to construct phenotype models of GMF from standardized data element concepts based on case definitions from the Gross Motor Function Classification System (GMFCS). Data concepts were selected using a theory and expert‐informed process and resulted in the construction of four phenotype models of GMF: an overall model and three classes corresponding to deviations in GMF for CP populations.

**Results:**

Sixty five data element concepts were identified for the overall GMF phenotype model. The 65 data elements correspond to 20 variables and logic statements that instantiate membership into one of three clinically meaningful classes of GMF. Data element concepts and variables are organized into five domains relevant to modeling GMF: Neurologic Function, Mobility Performance, Activity Performance, Motor Performance, and Device Use.

**Conclusion:**

Our experience provides an approach for organizations to leverage existing data for care improvement and research in other conditions. This is the first consensus‐based and theory‐driven specification of data elements and logic to support identification and labeling of GMF in patients for measuring improvements in care or the impact of new treatments. More research is needed to validate this phenotype model and the extent that these data differentiate between classes of GMF to support various LHS activities.

## 1 BACKGROUND

The re‐purposing of patient health data collected during routine patient care from the electronic health record (EHR) is more common over the past decade and can advance and support the formulation of real‐world knowledge, a key area of Learning Health Systems (LHS).^1^, ^2^, ^3^ Pediatric rehabilitation relies on EHR data to support clinical decision‐making of the interprofessional care team as well as LHS research and learning efforts to improve care delivery for patients with physical disability and deviations in functional performance. However, there is a paucity of systematic approaches to leverage EHR data in pediatric rehabilitation. Recent strategic plans by the National Institutes of Health (NIH) and National Institute for Child Health and Human Development (NICHD) emphasize building better rehabilitation research and learning infrastructure.^4^, ^5^ The re‐use of EHR data to characterize the range of patient functional performance through conceptual and digital phenotyping can support the evaluation of new and existing rehabilitative treatments and is of great value in rehabilitation settings. LHS infrastructure is designed to meet such needs across a broad range of health settings. However, in pediatric Cerebral Palsy (CP), there is no existing application, or systematic approach, for using EHR data to model complexity and deviations in physical functioning to support patient cohort identification.

Currently, few reports in the literature use analytic methods to “phenotype” patient cohorts in pediatric rehabilitation research, and only limited studies exist that develop or use typologies such as “phenotype models” from health data to characterize patient function in other settings.^6^, ^7^, ^8^, ^9^ A phenotype model contrasts a computable phenotype or phenotype algorithm, which are traditionally designed from EHR data elements and values and have computable rules dictated by patient data. Although a model is an informative representation of a system or person, a phenotype model is an informative representation of important and relevant data concepts that exist in an EHR. Fried et al^6^ describe a phenotype model in the context of frailty as a group of patient characteristics that, if present together, may represent a patient's level of frailty. Their model included variables for ambulation quality, reduced strength, unintentional weight loss, and reduced activity tolerance that were collected on patients aged 65 years or older in an observational cohort study.^6^, ^10^ Others have recently used the frailty phenotype model for a variety of applications, such as to support the construction of a frailty index based on the accumulation of deficits documented in an EHR to evaluate the extent of frailty in geriatric inpatients.^6^, ^7^, ^8^, ^9^ However, the absence of literature on phenotyping in rehabilitation research makes designing phenotype algorithms difficult because of the complexity of physical functioning. Hence, a phenotype model for functional performance is a critical infrastructure for LHSs in pediatric rehabilitation. Phenotyping approaches, such as Mo et al's desiderata for computable phenotyping using EHR data^11^ and others,^12^, ^13^, ^14^, ^15^ can be adapted to develop phenotype models of physical functioning from EHR data sources.

In the present study, a phenotype model structures key data concepts and value sets available in an EHR to characterize theoretical patient cohorts by deviations in functional performance, irrespective of the EHR data values. Analytical strategies, including phenotyping algorithms, to improve the identification of cohorts related to physical function would be a great benefit to research, quality improvement, and clinical practice in pediatric rehabilitation settings. Therefore, our work in developing a phenotype model of gross motor function (GMF) built on EHR data standards and architectures is more exploratory and conceptual and serves as a foundation for future phenotyping algorithms to define functional classes broadly from existing data sources.

### 1.1 Gross motor function classification system

Many clinicians (eg, orthopedic surgeons, physiatrists, occupational therapists (OT), physical therapists (PT), and nurse practitioners) use the Gross Motor Function Classification System (GMFCS) level to classify physical function during routine care for patients with CP. The GMFCS is a five‐level ordinal classification structure (ie, I, II, III, IV, V) and a standard screening tool used to classify deviations in the performance of GMF activities for children with CP.^16^, ^17^ Palisano et al^17^ illustrates these deviations and includes corresponding definitions. These deviations are frequently used in hip surveillance programs that focus on monitoring children with CP who may develop a hip dysplasia and subsequent displacement and dislocation.^18^, ^19^, ^20^, ^21^, ^22^, ^23^ However, the GMFCS is not always documented as a discrete data element in the EHR. Rather, it is often embedded in free‐text and dictated clinical notes using variations in terminologies, making EHR‐driven and automated cohort identification by functional performance levels more difficult.

GMFCS levels describe performance and participation rather than CP‐related physical impairment and body region involved (spastic hemiplegia, diplegia, tetraplegia, and quadriplegia).^18^ The GMFCS level definitions illustrate current functional status and have predictive value for a child's future functioning level with CP.^16^, ^17^, ^24^, ^25^ On one end, patients at GMFCS I are independent in all mobility activities and can run, jump, and play without physical limitations, and do not require the use of external devices. On the other end, patients at GMFCS V require total physical assistance to perform all activities, are unable to propel their own wheelchair, and require a manual wheelchair that is propelled by family or caregiver. The GMFCS is also divided into age‐ranges that reflect age‐related gross motor development and mobility skills (birth‐2, 2‐4, 4‐6, 6‐12, and 12‐18). These age‐range specific GMFCSs address similar underlying concepts in each case definition but are modified to reflect age‐appropriate activities. Although GMFCS level is considered stable after 2 years old,^16^, ^17^, ^25^ children generally achieve major gross motor developmental milestones by age 5.

### 1.2 Pediatric learning networks

In the past decade, federal funding and non‐profit organizations supported establishing LHS in pediatrics by developing several national clinical data research networks.^26^, ^27^, ^28^, ^29^, ^30^, ^31^, ^32^, ^33^, ^34^ PEDSnet, a Patient Centered Outcomes Research Institute (PCORI) funded effort, is one example of a general pediatric care learning network being used to support LHS activities.^29^, ^30^ The Shriners Hospitals for Children (SHC) Health Outcomes Network (SHOnet) is another learning network, one that is specific to the SHC system.^35^ SHOnet is the exemplary learning network for this use‐case and adapts the existing pediatric‐specific common data model (CDM) for PEDSnet built based on the Observational Medical Outcomes Partnership (OMOP) structure.^29^, ^30^, ^36^, ^37^ SHOnet harmonizes EHR data elements across 20 pediatric specialty hospitals in the SHC System. In addition to the OMOP concepts mapped in PEDSnet, the SHOnet CDM includes extensive mappings to EHR data elements for PT and OT observational discrete data elements. All SHOnet data elements for observational data and medications are stored as OMOP and RxNorm concept codes, respectively. This data infrastructure allows SHOnet to address many important treatment and research questions. In terms of the GMFCS values in SHOnet, due to EHR documentation practices at the clinician level, the GMFCS as a discrete data element has low completeness.^35^ The development of a phenotype model of GMF would build capacity to address questions related to functional outcomes stratified by functional performance levels.

## 2 STUDY OBJECTIVES

The overall aim of this study was to develop a methodology to build conceptual classification models of functional performance phenotypes from EHR data concepts in pediatric learning networks. Objective 1 of this aim was to construct a phenotype model of GMF using a theory and expert‐informed approach based on SHOnet CDM discrete data element concepts and using existing case definitions for each GMFCS level as gold‐standard phenotype definitions. Objective 2 of this aim was to define three clinically meaningful classes of GMF that were derived from an expert‐panel review of a set of data element concepts and corresponding value sets available in a pediatric EHR.

## 3 MATERIALS AND METHODS

### 3.1 Procedure

**FIGURE 1:**
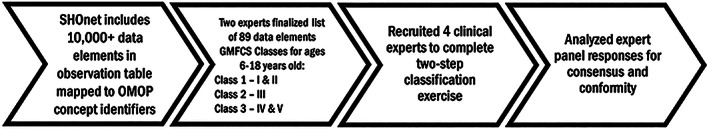
Flow diagram of the Gross Motor Function Phenotype Model development

The use of functional performance data elements to build patient cohorts for research or quality improvement is challenging because different clinicians observe and record physical functioning differently and this is not captured discretely or consistently by providers. Functional status can also represent a challenge because of the range of states (high functioning to low functioning), and it manifests differently in different patients. For this research, we designed a stepped and iterative process based on consensus expert review^38^ and adapted several methodologies^11^, ^12^, ^13^, ^14^, ^15^ to develop a phenotype model and corresponding subgroups, or classes, of GMF that uses theoretical data concepts from the SHOnet CDM. Figure 1 provides a flow diagram of our procedure to develop the GMF phenotype model (GMFPM).

Given this initial effort in what may be a more difficult classification, function across multiple subgroups rather than presence or absence of a condition on binary terms, for this study, the GMFCS was collapsed from five levels into three distinct classes of GMF. The three classes are largely consistent with major functioning levels: GMF Phenotype Class 1 includes GMFCS I and II, those who ambulate without assistive devices; GMF Phenotype Class 2 corresponds to GMFCS III, those individuals who use assistive devices including wheelchairs; GMF Phenotype Class 3 includes GMFCS IV and V, those individuals who have significant ambulatory limitations. Furthermore, all phenotype models in this study corresponded to patients aged 6‐18 years old because this is the largest age‐band of the GMFCS that overlaps with the school setting and clinical practice (eg, hip surveillance) guidelines and 6‐18‐year‐olds are expected to have stable GMFCS levels. The construction of the GMFPM and classes proceeded through four phases. As this model is foundational and is not a computable phenotype using real patient data, the validation of such a model is beyond the scope of this paper.

### 3.2 Four phases design process

In Phase 1, two SHOnet clinical domain experts, an OT and PT with experience treating patients with motor dysfunction, systematically selected data element concepts and value sets for panel review. These domain experts reviewed and scrutinized 10 000+ observational discrete data element concepts in the SHOnet CDM to be included for panel review. The initial set of data concepts were selected based on existing knowledge of routine care, evaluation, and treatment of patients with CP by SHC OTs, PTs, and nurses and if the concepts were thought to align with and deviate across GMFCS levels. For example, the data concept “Ambulation Level” had six possible values of mobility performance that demonstrate significant visible deviations between GMFCS levels and is known to be collected by OT and PT. Muscle tone and motor control dysfunction are common problems in patients with CP; therefore, data concepts for “Drooling” relates to oral motor control and concepts for “Elbow Tone” and “Ankle Tone” are known to deviate between GMFCS levels. Those not selected included data concepts for manual muscle testing of specific muscles and concepts on lab values that may be indeterminate of GMFCS levels. The initial set comprised 540 data element concepts. Domain experts then convened to review the initial selection of data concepts and removed those that were redundant or extraneous to functional performance. This resulted in a final selection of 89 data element concepts.

In Phase 2, the final set of data element concepts were consolidated into 31 unique and derived variables for use in the expert‐panel review exercise to assign and rate variables. A unique variable corresponds to one data element concept. A derived variable corresponds to many data element concepts that could be collapsed into one variable due to similarity in concept and value set. This also simplified the expert‐review process. For example, multiple data element concepts correspond to different types of assistive devices a patient may use with the same yes/no value set, so the concepts were combined to form the derived variable “Assistive Devices Used.” This contrasts the “Ambulation Level” variable, which is a single data element concept that maintains a standard 6‐level value set spanning “Independent” to “Dependent” performance. The 31 variables included 14 unique variables, 17 derived variables, and corresponding value sets. Variables and GMF classes formed a two‐step grid‐like exercise for expert‐panel review and completion.

**FIGURE 2:**
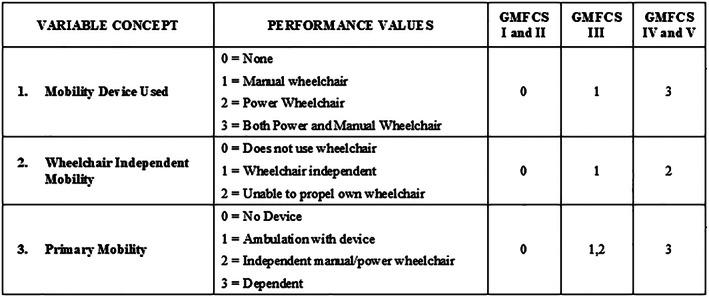
Example from EX1: Process for applying performance values for variables by each GMF class

**FIGURE 3:**
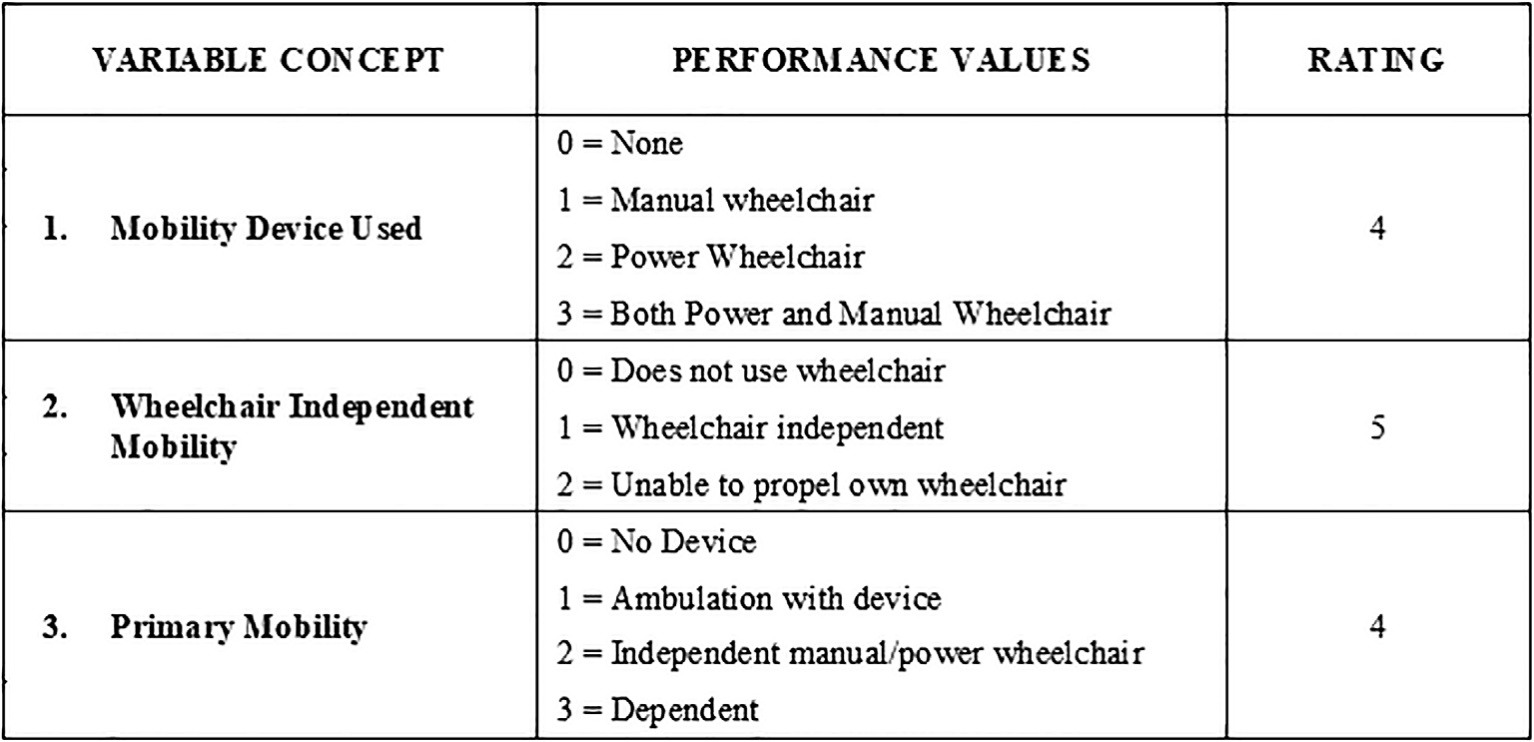
Example from EX2: Process for rating (on 5‐level scale) how well each variable differentiates between GMF classes

In Phase 3, we convened a panel of four new domain experts to support the design of the overall phenotype model and classes. The panel included four licensed clinicians and mobility researchers (three PTs and one OT) from three different SHCs with extensive knowledge of CP and an average of over 20 years of clinical experience. Panelists independently completed an evaluation exercise for the 31 variables by each GMF class. Figures 2 and 3 provide examples of the two‐step evaluation exercise (denoted EX1 and EX2). In EX1, panelists examined the extent that each of the 31 variables differentiated between the three GMF classes for patients 6 to 18 years old. For each GMF class, panelists assigned available performance values to each of the 31 variables, therefore each panelist completed 93 distinct classifications. If warranted, panelists assigned multiple values for each variable. In EX2, panelists rated their perception of how well variables distinguished between the three GMF classes by applying a 5‐point rating scale (1‐ does not distinguish at all, 3 ‐ distinguishes moderately, 5 ‐ distinguishes very well) to each variable. At the end of the exercise, panelists recommended additional variables they felt may distinguish between classes.

In Phase 4, we stratified the overall phenotype model by the three GMF classes based on the deviations in how panelists allocated performance values for all variables in EX1. The deviations in assigned values informed the construction of logic statements and rules for variable value sets to instantiate membership to one of the three GMF classes. Each statement contains human‐readable text and includes a stem, rule, and qualifier that is stratified by value sets for each variable. Two PhD trained, licensed PTs, on the SHOnet team with informatics and clinical domain expertise reviewed the structured rules, value assignments, and logical operators for variables in each GMF class for content validity.

### 3.3 Data analysis

The expert panel responses to the two‐step evaluation exercise were analyzed in three‐phases. First, we reviewed the values in EX1 that individual panelists assigned to the 31 variables across each GMF class and assigned an overall value per GMF class based on one of three results: (a) One variable value received panel consensus. (b) If panelists assigned multiple values to a variable or there was a tie in values, then both values comprised the final value for a GMF class (the inclusion of multiple values accounts for deviation in patient performance within a GMF class). (c) If the result in (a) or (b) did not occur, then the respective GMF classes included all values assigned by panelists.

Next, we analyzed the panel ratings in EX2 regarding the extent to which variables differentiated between the three GMF classes. The variables were analyzed for one of two criteria to be included in all GMF classes: (a) if a consensus of panelists rated the variable ≥3, or (b) if the panel responses for a variable were split, for example, if the variable received two ratings of ≤2 and two ratings of ≥3. Furthermore, the models did not include variables that received a consensus rating of ≤2.

In the final phase, we conducted a quality control check by analyzing the conformance between how panelists assigned values for each GMF class and how panelists rated the differentiation between GMF classes. Instances of conformance occurred when assigned variable values deviated across all GMF classes in EX1 and panelists rated the variable differentiation ≥3 in EX2. If the responses did not conform, then the variable was not included in the model. Microsoft Excel was used to complete all analyses.

## 4 RESULTS

**FIGURE 4:**
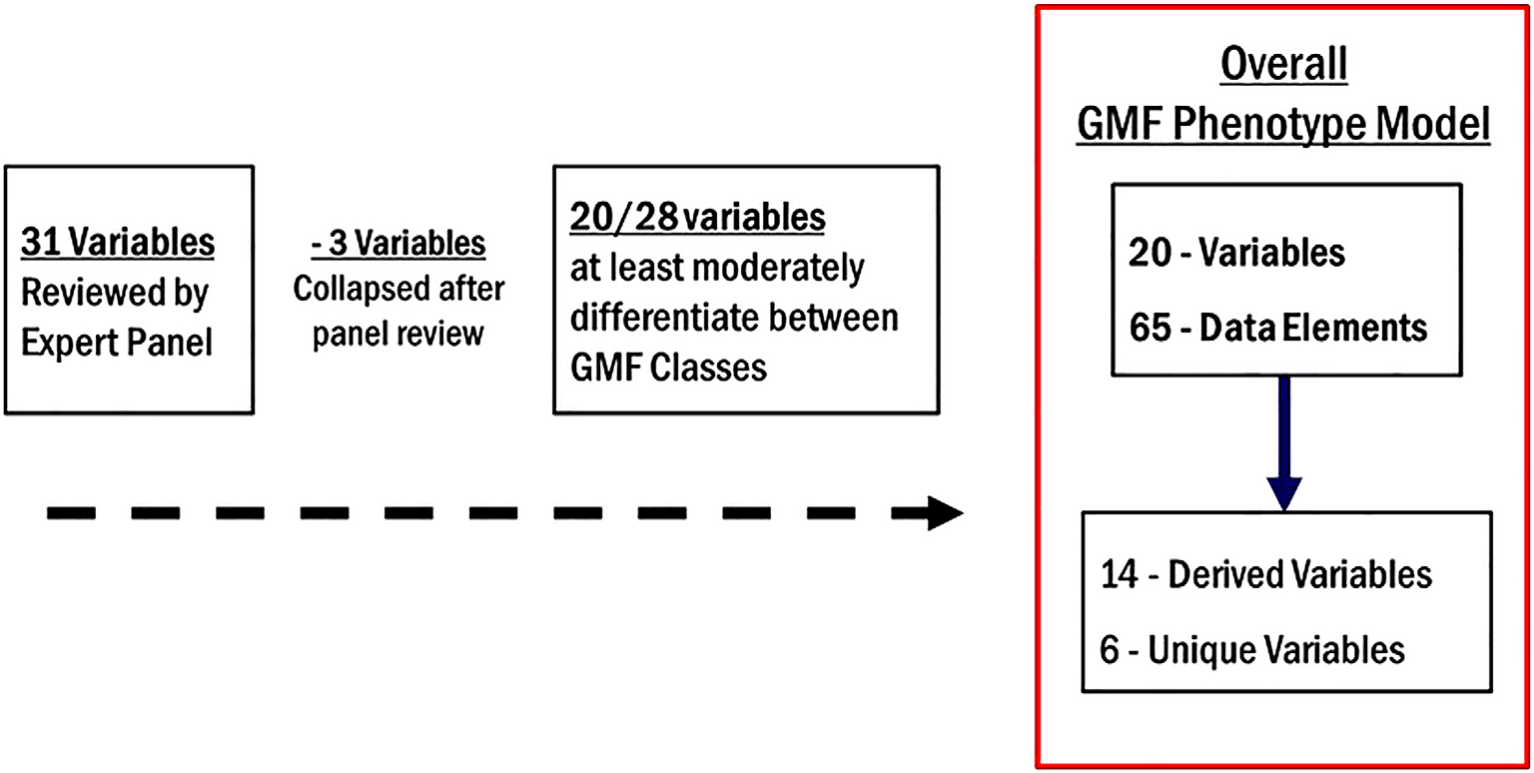
Results of evaluation of variables in the Gross Motor Function Phenotype Model

The overall GMFPM and three GMF classes included 20 variables that correspond to 65 performance‐related data elements that were identified by the expert panel. Table 1 provides a list of the 20 variables and 65 data elements and value sets in the overall phenotype model. Figure 4 provides a breakdown of the results for the overall phenotype model. The panel initially rated 23 of 31 variables to at least moderately (≥3) differentiate between GMF Phenotype classes; therefore, they agreed with approximately 73% of data element concepts selected by the SHOnet team. After the final analysis, three variables were added to existing variables to reduce negation in verbiage and redundancy and resulted in a total of 20 variables.

**TABLE 1:** Variables, data element concepts and value sets for each gross motor function domain

Domain	Variables	Data elements	Value set
Motor Performance	Drooling	Drooling; Drooling oral motor function	Yes/No
	Sitting Balance	Sitting balance	Intact; Impaired
	Knee Tone	Knee extensor tone; Knee flexor tone	0, 1, 1.5, 2, 3, 4
	Ankle Tone	Ankle dorsiflexor tone; Plantar flexor tone	0, 1, 1.5, 2, 3, 4
	Elbow Tone	Elbow Extensor Tone; Elbow flexor tone	0, 1, 1.5, 2, 3, 4
	Neck Strength	Neck Strength	WFL; Limited
Neurologic Function	Cognitive Concerns	Cognitive deficits; Speech deficit	Yes/No
	Communication Concerns	Language delay; Language impairment; Speech delay; Speech impairment	Yes/No
Devices Used	Assistive Devices Used	Walker; Cane; Crutches	Yes/No
	Mobility Device Used	Wheelchair independently; Manual wheelchair; Unable to propel own wheelchair; Power wheelchair	Yes/No
	Ambulation Device Used	Gait trainer; Swivel walker; Walker pickup; Walker reverse; Walker wheeled; Crutches forearm; Cane, quad; Cane, single point; Cane, tripod; Crutches axillary; Stander, None	Yes/No
	Stair Railings	Stair Railings	Bilateral; Rail on left going up; Rail on right going up; None
Activity Performance	Current Home Treatments	Respiratory support; Trach care; Tube feeding; Urinary catheterization	Yes/No
	Fine Motor Concerns	Dressing; Feeding; Grooming; Bathing	Yes/No
	Toileting Habits	Toilet trained; Diaper at night	Yes/No
	Feeding Ability	Feeds self; Complete independence; Modified independence; Supervision; Minimal assistance; Moderate Assistance; Maximal assistance; No oral feedings; Total assistance	Yes/No
Mobility Performance	Gross Motor Concerns	Ambulatory with assistance; Assistive devices needed; Household ambulation; Tires easily; Trips/falls frequently; Unable to sit independently; Non‐ambulatory	Yes/No
	Ambulation Level	Ambulation Level	Independent; Stand‐by assistance; Minimal assistance; Moderate assistance; Maximum assistance; Dependent
	Stairs Assistance	Stairs Assistance	Complete independence; Standby assistance; Contact guard assistance; Minimal assistance; Moderate assistance; Maximal assistance; Dependent
	Primary Mobility	Primary Mobility	Independent wheelchair –manual; Independent wheelchair –power; Ambulation with device; Ambulation without device; Dependent wheelchair mobility; Other

Variables were grouped into five performance‐related domains (Table 1) to organize and present the data concepts based on characteristics in the GMFCS definitions and components of Body Structures and Function, Activity, and Participation in the World Health Organization International Classification of Functioning, Disability and Health.^39^ The five domains include: Neurologic Function, Mobility Performance, Activity Performance, Motor Performance, and Device Use. In post‐exercise discussions, two panelists recommended additional data elements for gastrointestinal and anti‐epileptic/muscle relaxant medications because these medication types may help distinguish between high and low GMFCS levels. These corresponding RxNorm data concepts were not added to the model in this study.

**FIGURE 5:**
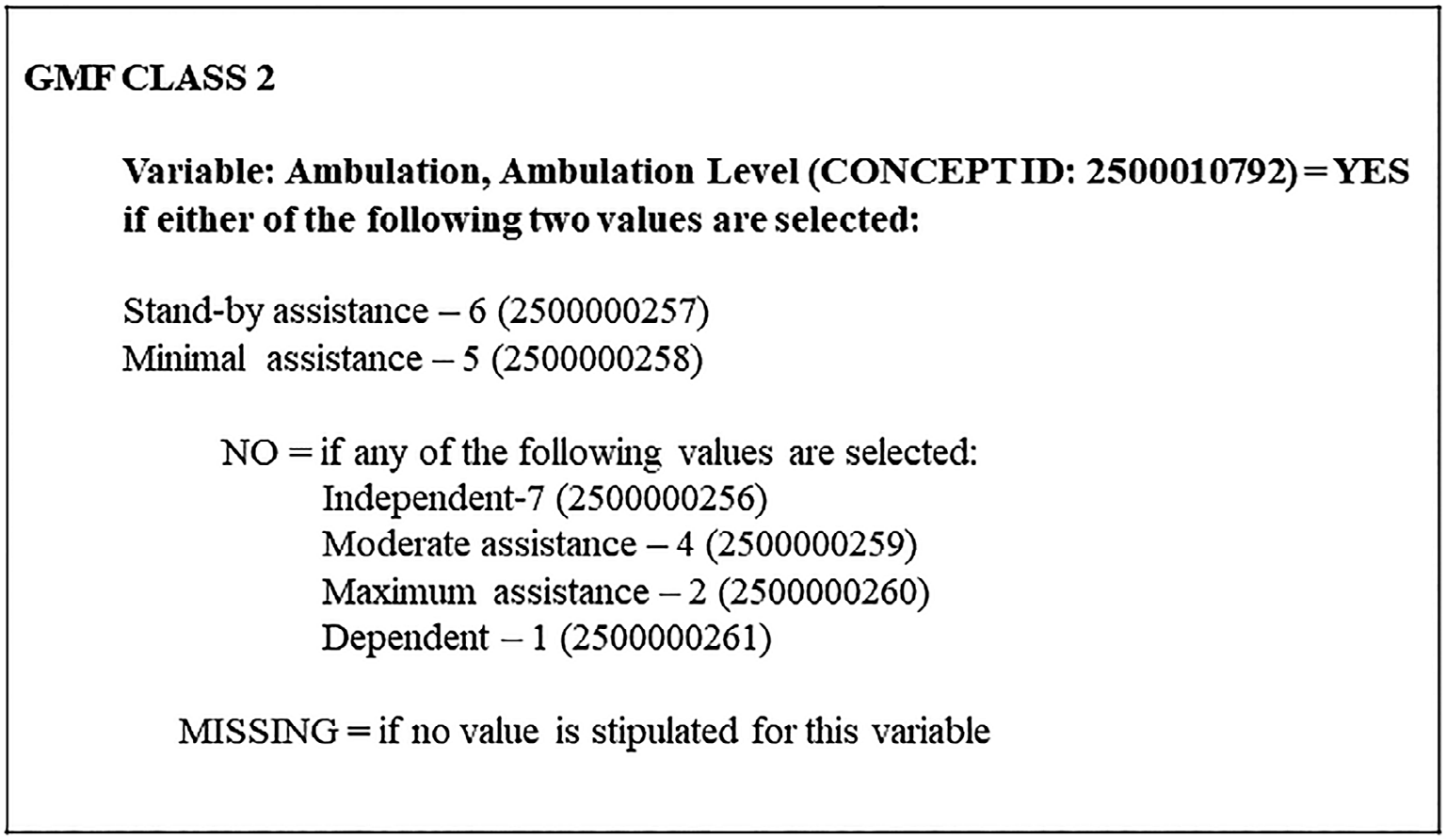
Logic statement and rule for Ambulation Level variable in GMF Class 2

Each GMF class comprised human readable logic statements and rules for each variable to instantiate membership to that respective GMF class. As an exemplar, Table 2 provides a matrix view of the Activity Performance domain and includes rules, variables, data element concepts, and value sets stratified by each GMF class. Figure 5 includes an example logic statement for the “Ambulation Level” variable to differentiate between GMF classes. The structured rules and logic statements for variables in each GMF class are included as Data S1. These statements include OMOP custom concept codes for data elements and value sets to encourage generalizability with other pediatric health system data warehouses, networks, and registries built using OMOP. The compilation of the structured rules provides an opportunity to study more granular deviations in physical functioning between GMFCS levels.

**TABLE 2:** Example: activity performance domain

Variable	Data Element Concepts	GMF Class 1	GMF Class 2	GMF Class 3
Current Home Treatments	Respiratory support (2500010257)	No treatments	No treatments	Any one treatment
	Trach care (2500010258)			
	Tube feeding (2500010259)			
	Urinary catheterization (2500010260)			
Fine Motor Concerns	Dressing (2500010181)	No fine motor concerns	Concerns with any one fine motor activity	More than one fine motor concern
	Feeding (2500010178)			
	Grooming (2500010182)			
	Bathing (2500010180)			
Toileting Habits	Toilet trained (2500000137)	Toilet Trained	Toilet trained or diaper/assistance	Diaper at night, assistance needed
	Diaper at night (2500000135)			
Feeding Ability	Feeds self (2500000144)	Feeds self, Independence	Feeds self, Independence, Supervision, Minimum to Maximum Assistance	Minimum to Total Assist, or No Oral Feedings
	Complete Independence (2500000143)			
	Modified independence (2500000148)			
	Supervision (2500000150)			
	Minimal Assistance (2500000146)			
	Moderate Assistance (2500000147)			
	Maximal Assistance (2500000145)			
	No oral feedings (2500000149)			
	Total assistance (2500000151)			

### 4.1 Analysis of conformance

The analysis of conformance revealed inconsistencies for two variables. The first variable was “General Lower Extremity Muscle Tone”. Although panelists perceived that this variable moderately differentiated (≥3) between GMF classes, the panelists all assigned the same performance value to each GMF class. Due to the inconsistency, this variable was not included in the overall model. This may have occurred due to the decreased granularity in the value set for this variable. Fortunately, the panel also selected two other lower extremity derived variables that were joint‐specific and had more granular value sets: “Knee Tone” and “Ankle Tone.” These variables included both flexor and extensor tone data elements and are scored using the Modified Ashworth Scale (MAS). The MAS is a standardized 6‐level ordinal scale (ie, 0, 1, +1, 2, 3, 4) of muscle tone and better deviates between GMF classes compared to variables for presence of general tone with yes/no value sets.

The second inconsistency occurred for the variable “Elbow Tone.” On the 5‐level rating of differentiation between GMF classes, two panelists rated “Elbow Tone” ≤2, one panelist declined to rate the variable, and another rated the variable a 3. The overall performance values applied to “Elbow Tone” using the MAS followed a clear gradation across each GMF class [(MAS scores: GMF Class 1:0,1,1+; GMF Class 2:1,1+, 2,3; GMF Class 3:2,3,4)]. At the discretion of SHOnet domain experts, “Elbow Tone” was included in all models.

## 5 DISCUSSION

In this study, we developed a standards‐based, expert‐informed GMFPM that also offers flexibility across three clinically meaningful classes aligned with the GMFCS. This is the first instance where CDM data concepts are organized into a phenotype model of functional performance specifically for pediatric rehabilitation. The findings illustrate that GMF for initial cohort identification activities in pediatric rehabilitation can be represented through phenotypes of discrete data elements within standardized database models used widely in CDMs for learning networks, data registries, and data warehouses. This study also underscores the complexity of modeling functional performance using standardized data elements and the rigor necessary to develop similar typologies of functional performance in the future; therefore, our methods should be informative, nonetheless.

The work supports future infrastructure by exploring what data are available that fit into a conceptual model (GMFPM) that the CP community has already decided are important. These data element concepts can later be leveraged for different applications, including the development of a phenotype algorithm that can be deployed and validated on existing data for a given purpose. Our phenotype model will undergo additional study and validation to determine its performance in differentiating between GMF classes. In addition, this phenotype model serves as a measurement instrument to determine the documentation and value of these EHR data elements.

The rigorous approach to construct the phenotype model and classes was contextualized by the desiderata for computable phenotyping and information modeling work by Westra et al.^11^, ^38^ Westra et al^38^ developed an information model of structured flowsheet data elements to support secondary data use in health systems research. However, their work uses a data‐driven consensus process informed by the available structured data values across a large hospital system. This contrasts our study which used an initial theory‐driven approach to compile data element concepts that support semantic interoperability, followed by an expert‐informed consensus‐based review and analysis of conformance.^38^ In addition, Morley et al^12^ demonstrate the use of expert‐panel review for developing phenotyping algorithms; however, our focus on physical function and a multi‐step rating process by the panel highlights significant differences between phenotyping algorithms and developing conceptual phenotype models using CDM data concepts. Much like Westra, the design of this phenotype model helps simplify the representation of CDM data concepts for specific research and evaluation purposes that use EHR data.^38^ However, though pragmatic, the drawback in using the data element values that providers document in the EHR to construct typologies such as ours is that these data perpetuate bias from clinical documentation practices and the selection of data elements providers use for clinical documentation.^40^

The methods described in this paper support the theory‐based selection of data elements and corresponding interface terminologies to design the logic statements and rules capable of classifying cohorts of patients by deviations in GMF. The underlying rationales for provider documentation are unknown, but discrete data are likely documented for billing and administrative purposes, while diagnostic reasoning, including GMFCS levels and other information used to form these clinical phenotypes, is included in narrative documentation. Nevertheless, our findings demonstrate the inherent value of designing function‐based mechanisms around discrete data elements readily available in an EHR and learning network and emphasizes the need for improved capture of meaningful and usable clinical data. The benefit of using a theory‐driven, expert‐informed approach is that the typologies are not constrained by what data *are collected*. Instead, the typologies may be interoperable with other standards‐based pediatric data resources and foregrounds what data *should be collected* by clinicians and systems to classify clinically sensible classes of GMF. Moreover, the iterative approach we used demonstrates the utility of stretching existing methodologies into developing “functional” phenotypes for pediatric rehabilitation.

This expert‐informed GMFPM may support future predictive analytics of GMFCS for research; however, this study has strengths and limitations. The primary limitation of this study is its generalizability. Since SHC is a specialty pediatric healthcare system, the documentation of many of these data elements and their inclusion in SHOnet may be different from other systems. Other pediatric healthcare systems that manage general pediatric disorders may not prioritize, document, or have fields in the EHR for many of the data elements in the phenotype model.

In terms of strengths, our study devised and applied a foundational methodological approach to phenotyping that could very easily be adapted to any other use cases, particularly in the field of medical rehabilitation. Evidence demonstrates that the re‐use of EHR data improves patient cohort identification and may be essential to support pragmatic prospective cohort studies with the economy of scale.^6^, ^7^, ^8^, ^9^, ^41^, ^42^, ^43^, ^44^ However, the discrepancy between derived definitions and the performance and use of phenotypes in practice points to a need to improve the identification and agreement of clinical characteristics in EHR‐based phenotypes.^41^ The methodological approach and use of data concepts from a CDM described in this study helps fill this gap. A significant strength of the study was that it used data element concepts based on a standardized terminology of medical concepts (ie, OMOP). OMOP includes widely accepted reference terminology standards and publicly available concept codes which further supports opportunities for generalized use. Another strength of this study was the investigator blinding to completeness or availability of patient EHR data in the initial review and selection process because this knowledge could have biased the theory‐based selection of data element concepts. Lastly, panelists all worked at three regionally different SHCs; therefore, the regional variation and priorities in practice may mitigate potential biases in their ratings.

The GMFPM, although not operational in an EHR, builds infrastructure from a CDM to identify pediatric patient cohorts by distinct categories of GMF for research and quality improvement. Our findings can also inform other multi‐site research and learning networks that support pediatric populations (ie, PEDSnet, ImproveCareNow) of the opportunities afforded by building out their data elements for measurement infrastructure to conduct critical LHS research in rehabilitation. Future work should analyze data quality dimensions of the phenotype model, the extent that the typologies can validly differentiate between GMF classes, and its utility in applications such as CP hip surveillance efforts. More use‐cases of phenotypes for characterizing functional performance and care processes are needed to build a computable measurement library with economy of scale and scope for pediatric rehabilitation.

## CONFLICT OF INTEREST

The authors have no conflict of interest to disclose.

## Supporting information


**Data S1.** Supporting Information.
