# Supplementary material for: Development of a standards‐based phenotype model for gross motor function to support learning health systems in pediatric rehabilitation
Source: Learn Health Syst. 2021 May 5;6(1):e10266. doi: 10.1002/lrh2.10266 (PMC8753308; doi:10.1002/lrh2.10266)
Supplement: Supplementary file 1 — Data S1. Supporting Information. [file LRH2-6-e10266-s001.docx]

Structured Rules of the Gross Motor Function Phenotype Classes

**GMF CLASS 1 (GMFCS 1 & 2)**

**The following rules comprise derived variables composed of data elements and their value sets to satisfy the rules.**

**ACTIVITIES DOMAIN 19 data elements, 4 variables**

**Derived variable: Current Home Treatments = YES if all 4 variables below are NO:**

Respiratory support (2500010257)

Trach care (2500010258)

Tube feeding (2500010259)

Urinary catheterization (2500010260)

NO = If any variable is stipulated as YES

MISSING = If all the variables are MISSING

**Derived variable: Fine Motor Concerns (CONCEPT ID: 2500010797; Qualifier: 2500000275) = YES if all the following four variables are any combination of NO or MISSING:**

Dressing (2500010181)

Feeding (2500010178)

Grooming (2500010182)

Bathing (2500010180)

NO = if at least one of the four variables are stipulated as YES

**Derived variable: Toileting habits (CONCEPT ID: 2500010700) = YES if the following variable is YES:**

Toilet trained (2500000137)

**AND** the following variable is NO:

Diaper at night (2500000135)

NO = if the following variable is YES:

Diaper at night (2500000135)

MISSING = If “Diaper at night” is NO AND “Toilet trained” is MISSING

**Derived variable: Nutritional Screen, Feeding Ability (CONCEPT ID: 2500010703) = YES if any one of the following variables is YES:**

Feeds self (2500000144)

Complete Independence (2500000143)

Modified independence (2500000148)

**AND** any of the following variables are NO:

Total assistance (2500000151)

Maximal Assistance (2500000145)

No oral feedings (2500000149)

Minimal Assistance (2500000146)

Moderate Assistance (2500000147)

Supervision (2500000150)

NO = if all the following variables are NO:

Feeds self (2500000144)

Complete Independence (2500000143)

Modified independence (2500000148)

**AND** any of the following variables are YES:

Total assistance (2500000151)

Maximal Assistance (2500000145)

No oral feedings (2500000149)

Minimal Assistance (2500000146)

Moderate Assistance (2500000147)

Supervision (2500000150)

MISSING = if all the following variables are MISSING:

Feeds self (2500000144)

Complete Independence (2500000143)

Modified independence (2500000148)

**AND** all the following variables are NO or MISSING:

Total assistance (2500000151)

Maximal Assistance (2500000145)

No oral feedings (2500000149)

Minimal Assistance (2500000146)

Moderate Assistance (2500000147)

Supervision (2500000150)

**NEUROLOGICAL DOMAIN 6 data elements, 2 variables**

**Derived variable: Current Sensory Deficits = YES if both variables below are NO:**

Cognitive deficits (443432)

Speech deficit (432730)

NO = If one variable is stipulated as YES

MISSING = If both variables are MISSING

**Derived Variable: Communications Concerns (CONCEPT ID: 2500010797; Qualifier: 2500000271) = YES if all of the four below are NO:**

Language delay (4039748)

Language impairment (4041822)

Speech delay (4047123)

Speech impairment (435642)

NO = If any of the four are stipulated as YES

MISSING = if one of the four are missing AND the others coded as NO

**MEDICATIONS DOMAIN 17 data elements, 2 variables**

**Derived variable: Gastrointestinal medications = YES if none of the medications below are presently prescribed:**

Ranitidine (Drug Concept ID: 961047, 19126405, 961168, 19003290)

Famotidine (Drug Concept ID: 953076, 19077241, 19021074, 19027493, 953102)

NO = If any are presently prescribed

**Derived variable: Seizure/Muscle Relaxant medications = YES if none of the medications below are presently prescribed:**

Diazepam (Drug Concept ID: 19076372, 723013, 19018909, 19076374, 723042, 723020)

Valproic acid (Drug Concept ID: 40237988, 40238017)

NO = If any are presently prescribed

**DEVICES DOMAIN 19 data elements, 3 variables**

**Derived Variable: Assistive Devices Used = YES if all of the following variables are NO or MISSING, or one of the following three variables are YES**:

Cane (4337514)

Crutches (4179721)

Walker (45767825)

NO = if two or more of the following variables is YES:

Cane (4337514)

Crutches (4179721)

Walker (45767825)

**Derived variable: Mobility Device Used = YES if the all the following four variables are any combination of NO or MISSING**:

Wheelchair independently (4012670)

Manual wheelchair (4045112)

Power wheelchair (2616920)

Unable to propel own wheelchair (4215087)

NO = if any variable is stipulated as YES

**Derived variable: Ambulation Device Utilized = YES if any one of the following 7 variables are YES:**

None (2500010164)

Cane, quad (2500010160)

Cane, single point (2500010161)

Cane, tripod (2500010162)

Crutches axillary (45772311)

Crutches forearm (2616479)

Walker wheeled (2616497)

Gait trainer (3038446)

Stander (37396481)

Swivel walker (45764219)

Walker pickup (2616486)

Walker reverse (2500010165)

NO = if two or more of the following variables are stipulated YES:

Cane, quad (2500010160)

Cane, single point (2500010161)

Cane, tripod (2500010162)

Crutches axillary (45772311)

Crutches forearm (2616479)

Walker wheeled (2616497)

Gait trainer (3038446)

Stander (37396481)

Swivel walker (45764219)

Walker pickup (2616486)

Walker reverse (2500010165)

MISSING = if ALL the following variables are MISSING:

None (2500010164)

Cane, quad (2500010160)

Cane, single point (2500010161)

Cane, tripod (2500010162)

Crutches axillary (45772311)

Crutches forearm (2616479)

Stander (37396481)

Gait trainer (3038446)

Swivel walker (45764219)

Walker pickup (2616486)

Walker reverse (2500010165)

Walker wheeled (2616497)

**MOBILITY DOMAIN 11 data elements, 5 variables**

**Derived Variable: Gross Motor Concerns (CONCEPT ID: 2500010797; Qualifier: 2500010797) = YES if the all of the following variable is NO or one or more is YES:**

Ambulatory with assistance (2500010171)

Tires easily (45881740)

Household ambulation (2500010186)

Assistive devices needed (3039217)

Trips/falls frequently (2500010215)

**AND** the following variables are NO:

Unable to sit independently (4106332)

Non-ambulatory (2500010193)

NO = If at least one of the following two is YES:

Unable to sit independently (4106332)

Non-ambulatory (2500010193)

MISSING = if “Unable to sit independently (4106332) and Non-ambulatory (2500010193) NO or MISSING and all other variables in this derived variable MISSING.

**Variable: Ambulation, Ambulation Level (CONCEPT ID: 2500010792) = YES if the following value is selected:**

Independent-7 (2500000256)

NO = if any of the following values are selected:

Stand-by assistance – 6 (2500000257)

Minimal assistance – 5 (2500000258)

Moderate assistance – 4 (2500000259)

Maximum assistance – 2 (2500000260)

Dependent – 1 (2500000261)

MISSING = if no value is stipulated for this variable

**Variable: Stairs Assistance (CONCEPT ID: 2500010796) = YES if any of the following three values are selected:**

Complete independence (2500000143)

Standby assistance (2500000269)

NO = if any of the following values are selected:

Contact guard assistance (2500000270)

Minimal assistance (2500000146)

Moderate Assistance (2500000147)

Maximal Assistance (2500000145)

Dependent (4159760)

MISSING = if no value is stipulated for this variable

**Variable: Ambulation Railings (CONCEPT ID: 2500010795) = YES if any one of the following three values below is selected:**

Rail on left going up (2500000267)

Rail on right going up (2500000268)

None (4124462)

NO = If the following value is selected

Bilateral (2500000266)

MISSING = if no value is stipulated AND stairs assistance variable has a value

**Variable: Primary Mobility (CONCEPT ID:2500000268) = YES if one of the following two values is selected:**

Ambulation without device (2500000358)

NO = if any of the following values is selected:

Ambulation with device (2500000357)

Dependent wheelchair mobility (2500000359)

Independent wheelchair mobility – manual (2500000360)

Independent wheelchair mobility – power (2500000361)

Other (9177)

MISSING = if no value is stipulated for this variable

**MOTOR DOMAIN 7 data elements, 6 variables**

**Derived Variable: Drooling = YES if either of the following variables is NO or MISSING:**

Nutritional Risk Factors (CONCEPT ID: 2500010706): Drooling (2500000178)

Drooling Oral Motor Function (28312333)

NO = if either of the following variables is YES

Nutritional Risk Factors (CONCEPT ID: 2500010706): Drooling (2500000178)

Drooling Oral Motor Function (28312333)

**Variable: Sitting Balance PT (4186717, (Source Value 28295811)) = YES if the following value is selected:**

Intact

NO if the following value is selected:

Impaired

MISSING = if no value is stipulated for this variable

**Variable: Knee Flexors/Extensors** **(2500010279/2500010278) = YES if the following values are selected on the Modified Ashworth Scale:**

0

1

1.5

NO = if one of the following values is selected:

2

3

4

MISSING = if no value is stipulated in this variable

**Variable: Ankle Dorsiflexors/Plantar flexors** **(2500010262/2500010265) = YES if the following values are selected on the Modified Ashworth Scale:**

0

1

1.5

NO = if one of the following values is selected:

2

3

4

MISSING = if no value is stipulated in this variable

**Variable: Elbow flexor/extensor muscle tone (2500010267/2500010266) = YES if the following values are selected on the Modified Ashworth Scale:**

0

1

1.5

NO = if one of the following values is selected:

2

3

4

MISSING = if no value is stipulated in this variable

**Variable: General Strength, Neck (2500010813) =YES if the following value is selected:**

WFL (2500000041)

NO = if the following value is selected:

Limited (2500000040)

MISSING = if no value is stipulated in this variable

**GMF CLASS 2 (GMFCS 3)**

**The following rules comprise derived variables composed of data elements and their value sets to satisfy the rules.**

**ACTIVITIES DOMAIN 19 data elements, 4 variables**

**Derived variable: Current Home Treatments = YES if all the following 4 variables below are NO:**

Respiratory support (2500010257)

Trach care (2500010258)

Tube feeding (2500010259)

Urinary catheterization (2500010260)

NO = If any of the variables are stipulated as YES

MISSING = If one variable is stipulated as NO AND one or more variables are MISSING

**Derived variable: Fine Motor Concerns (CONCEPT ID: 2500010797; Qualifier: 2500000275) = YES if one or more of the following four variables is YES:**

Dressing (2500010181)

Feeding (2500010178)

Grooming (2500010182)

Bathing (2500010180)

NO = if all four are stipulated as NO

MISSING = if any of the four variables are MISSING with one coded as NO

**Derived variable: Toileting habits (CONCEPT ID: 2500010700) = YES if one of the following variables is YES:**

Toilet trained (2500000137)

Diaper at night (2500000135)

MISSING = If no variable value is stipulated

**Derived variable: Nutritional Screen, Feeding Ability (CONCEPT ID: 2500010703) = YES if any one of the following variables is YES:**

Feeds self (2500000144)

Complete Independence (2500000143)

Modified independence (2500000148)

Supervision (2500000150)

Minimal Assistance (2500000146)

**AND** any of the following variables are NO:

Moderate Assistance (2500000147)

Maximal Assistance (2500000145)

No oral feedings (2500000149)

Total assistance (2500000151)

NO = if all the following variables are NO:

Feeds self (2500000144)

Complete Independence (2500000143)

Modified independence (2500000148)

Supervision (2500000150)

Minimal Assistance (2500000146)

**AND** any of the following variables are YES:

Moderate Assistance (2500000147)

Maximal Assistance (2500000145)

No oral feedings (2500000149)

Total assistance (2500000151)

MISSING = if all the following variables are MISSING:

Complete Independence (2500000143)

Feeds self (2500000144)

Modified independence (2500000148)

Supervision (2500000150)

Minimal Assistance (2500000146)

**AND** all the following variables are NO or MISSING:

Moderate Assistance (2500000147)

Maximal Assistance (2500000145)

No oral feedings (2500000149)

Total assistance (2500000151)

**NEUROLOGIC DOMAIN 6 data elements, 2 variables**

**Derived variable: Cognitive Concerns = YES if one of the two below is YES:**

Cognitive deficits (443432)

Speech deficit (432730)

NO = If both are stipulated as NO

MISSING = If one variable is missing AND the other coded as NO

**Derived Variable: Communications Concerns (CONCEPT ID: 2500010797; Qualifier: 2500000271) = YES if all of the following variables are either NO or one of the four below is YES:**

Language delay (4039748)

Language impairment (4041822)

Speech delay (4047123)

Speech impairment (435642)

NO = If two or more of the four are stipulated as NO

MISSING = if any of the four variables are missing AND the others coded as NO

**MEDICATIONS DOMAIN 17 data elements, 2 variables**

**Derived variable: Gastrointestinal medications = YES if none of the medications below are presently prescribed:**

Ranitidine (Drug Concept ID: 961047, 19126405, 961168, 19003290)

Famotidine (Drug Concept ID: 953076, 19077241, 19021074, 19027493, 953102)

NO = If any are presently prescribed

**Derived variable: Seizure/Muscle Relaxant medications = YES if none of the medications below are presently prescribed:**

Diazepam (Drug Concept ID: 19076372, 723013, 19018909, 19076374, 723042, 723020)

Valproic acid (Drug Concept ID: 40237988, 40238017)

NO = If any are presently prescribed

**DEVICES DOMAIN 20 data elements, 4 variables**

**Derived Variable: Assistive Devices Used = YES if one or more of the following variables is YES**:

Walker (45767825)

Cane (4337514)

Crutches (4179721)

NO = if all the variables are stipulated as NO

MISSING = if no variables values are stipulated

**Derived variable: Mobility Device Used = YES if the following variable is YES**:

Wheelchair independently (4012670)

**AND** the following variables is YES:

Manual wheelchair (4045112)

**AND** the following variable is NO or MISSING:

Unable to propel own wheelchair (4215087)

Power wheelchair (2616920)

NO = if “Unable to propel own wheelchair” or “Power Wheelchair” are YES and “Wheelchair independently” is NO

MISSING = if “wheelchair independently” is MISSING and “unable to propel own wheelchair” is NO; OR if all variables are MISSING

**Derived variable: Ambulation Device Utilized = YES if one or more of the following 6 variables are YES:**

Gait trainer (3038446)

Swivel walker (45764219)

Walker pickup (2616486)

Walker reverse (2500010165)

Walker wheeled (2616497)

Crutches forearm (2616479)

Cane, quad (2500010160)

Cane, single point (2500010161)

Cane, tripod (2500010162)

Crutches axillary (45772311)

Stander (37396481)

NO = if any of the following variables are stipulated YES:

None (2500010164)

MISSING = if ALL the following variables are MISSING and None is NO:

Gait trainer (3038446)

Swivel walker (45764219)

Walker pickup (2616486)

Walker reverse (2500010165)

Walker wheeled (2616497)

Crutches forearm (2616479)

Cane, quad (2500010160)

Cane, single point (2500010161)

Cane, tripod (2500010162)

Crutches axillary (45772311)

Stander (37396481)

**Variable: Ambulation Railings (CONCEPT ID: 2500010795) = YES if one of the following three values below is selected:**

Bilateral (2500000266)

Rail on left going up (2500000267)

Rail on right going up (2500000268)

NO = if the following value is selected:

None (4124462)

MISSING = if no value is stipulated for this variable AND stairs assistance variable has a value

**MOBILITY DOMAIN 10 data elements, 4 variables**

**Derived Variable: Gross Motor Concerns (CONCEPT ID: 2500010797; Qualifier: 2500010797) = YES if one or more of the following variables is YES:**

Ambulatory with assistance (2500010171)

Assistive devices needed (3039217)

Household ambulation (2500010186)

Tires easily (45881740)

Trips/falls frequently (2500010215))

**AND** the following variables are NO:

Unable to sit independently (4106332)

Non-ambulatory (2500010193)

NO = If all the following are NO or MISSING:

Ambulatory with assistance (2500010171)

Assistive devices needed (3039217)

Household ambulation (2500010186)

Tires easily (45881740) [AND/OR]

Trips/falls frequently (2500010215))

**AND** one or more of the following two is YES:

Unable to sit independently (4106332)

Non-ambulatory (2500010193)

MISSING = if “Unable to sit independently” AND “Non-ambulatory” are MISSING or NO and all other variables in this derived variable are NO or MISSING

**Variable: Ambulation, Ambulation Level (CONCEPT ID: 2500010792) = YES if either of the following two values are selected:**

Stand-by assistance – 6 (2500000257)

Minimal assistance – 5 (2500000258)

NO = if any of the following values are selected:

Independent-7 (2500000256)

Moderate assistance – 4 (2500000259)

Maximum assistance – 2 (2500000260)

Dependent – 1 (2500000261)

MISSING = if no value is stipulated for this variable

**Variable: Stairs Assistance (CONCEPT ID: 2500010796) = YES if any of the following five values are selected:**

Standby assistance (2500000269)

Contact guard assistance (2500000270)

Minimal assistance (2500000146)

Moderate Assistance (2500000147)

Maximal Assistance (2500000145)

NO = if any of the following values are selected:

Complete independence (2500000143)

Dependent (4159760)

MISSING = if no value is stipulated for this variable

**Variable: Primary Mobility (CONCEPT ID:2500000268) = YES if one of the following three values is selected:**

Independent wheelchair mobility – manual (2500000360)

Independent wheelchair mobility – power (2500000361)

Ambulation with device (2500000357)

NO = if any of the following values is selected:

Ambulation without device (2500000358)

Dependent wheelchair mobility (2500000359)

Other (9177)

MISSING = if no value is stipulated for this variable

**MOTOR DOMAIN 10 data elements, 6 variables**

**Derived Variable: Drooling = YES if either of the following variables is NO or MISSING:**

Nutritional Risk Factors (CONCEPT ID: 2500010706): Drooling (2500000178)

Drooling Oral Motor Function (28312333)

NO = if one of the above variables is YES

**Variable: Sitting Balance PT (4186717, (Source Value 28295811)) = YES if the following value is selected:**

Intact

NO = if the following value is selected:

Impaired

MISSING = if no value is stipulated for this variable

**Derived Variable: Knee Flexor/Extensor Tone** **(2500010279/2500010278) = YES if one of the following values are selected on the Modified Ashworth Scale:**

1

1.5

2

3

NO = if one of the following values is selected:

0

4

MISSING = if no value is stipulated in this variable

**Derived Variable: Ankle Dorsiflexors/Plantar flexors** **(2500010262/2500010265) = YES if one of the following values are selected on the Modified Ashworth Scale:**

1

1.5

2

3

NO = if one of the following values is selected:

0

4

MISSING = if no value is stipulated in this variable

**Derived Variable: Elbow flexor/extensor muscle tone (2500010267/2500010266) = YES if the following values are selected on the Modified Ashworth Scale for either of the above variables:**

1

1.5

2

3

NO = if one of the following values is selected:

0

4

MISSING = if no value is stipulated in this variable

**Variable: General Strength, Neck (2500010813) =YES if the following value is selected:**

WFL (2500000041)

NO = if the following value is selected:

Limited (2500000040)

MISSING = if no value is stipulated in this variable

**GMF CLASS 3 (GMFCS 4 & 5)**

**The following rules comprise derived variables composed of data elements and their value sets to satisfy the rules**

**ACTIVITIES DOMAIN 19 data elements, 4 variables**

**Derived variable: Current Home Treatments = YES if one or more of the four below is YES:**

Respiratory support (2500010257)

Trach care (2500010258)

Tube feeding (2500010259)

Urinary catheterization (2500010260)

NO = If all four are stipulated as NO

MISSING = missing if any of the four are missing with the others coded as NO

**Derived variable: Fine Motor Concerns (CONCEPT ID: 2500010797; Qualifier: 2500000275) = YES if two or more of the following four variables is YES:**

Dressing (2500010181)

Feeding (2500010178)

Grooming (2500010182)

Bathing (2500010180)

NO = if three or more are stipulated as NO

MISSING = if any of the four are MISSING with one coded as NO

**Derived variable: Toileting habits (CONCEPT ID: 2500010700) = YES if the convention below is satisfied:**

Diaper at night (2500000135) = YES

**AND** the following variable is NO**:**

Toilet trained (2500000137)

NO = if “Toilet trained” is YES

MISSING = if “Diaper at night” is missing “Toilet trained” is NO

**Derived variable: Nutritional Screen, Feeding Ability (CONCEPT ID: 2500010703) = YES if any one of the following variables is YES:**

Minimal Assistance (2500000146)

Moderate Assistance (2500000147)

Maximal Assistance (2500000145)

Total assistance (2500000151)

No oral feedings (2500000149)

**AND** any of the following variables are NO or MISSING:

Feeds self (2500000144)

Complete Independence (2500000143)

Modified independence (2500000148)

Supervision (2500000150)

NO = if all the following variables are MISSING or NO:

Minimal Assistance (2500000146)

Moderate Assistance (2500000147)

Maximal Assistance (2500000145)

Total assistance (2500000151)

No oral feedings (2500000149)

**AND** any of the following variables are YES:

Feeds self (2500000144)

Complete Independence (2500000143)

Modified independence (2500000148)

Supervision (2500000150)

MISSING = if all the following variables are MISSING

Minimal Assistance (2500000146)

Moderate Assistance (2500000147)

Maximal Assistance (2500000145)

Total assistance (2500000151)

No oral feedings (2500000149)

**AND** all the following variables are NO or MISSING:

Feeds self (2500000144)

Complete Independence (2500000143)

Modified independence (2500000148)

Supervision (2500000150)

**NEUROLOGICAL DOMAIN 6 data elements, 2 variables**

**Derived variable: Current Sensory Deficits = YES if one of the two below is YES:**

Cognitive deficits (443432)

Speech deficit (432730)

NO = If both are stipulated as NO

MISSING = missing if either are missing with the others coded as NO

**Derived Variable: Communications Concerns (CONCEPT ID: 2500010797; Qualifier: 2500000271) = YES if two or more of the four below is YES:**

Language delay (4039748)

Language impairment (4041822)

Speech delay (4047123)

Speech impairment (435642)

NO = If all four are stipulated as NO

MISSING = missing if any of the four are missing with the others coded as NO

**MEDICATIONS DOMAIN 17 data elements, 2 variables**

**Derived variable: Gastrointestinal medications = YES if one or more of the medications below are presently prescribed:**

Ranitidine (Drug Concept ID: 961047, 19126405, 961168, 19003290)

Famotidine (Drug Concept ID: 953076, 19077241, 19021074, 19027493, 953102)

NO = If any are presently prescribed

**Derived variable: Seizure/Muscle Relaxant medications = YES if one or more of the medications below are presently prescribed:**

Diazepam (Drug Concept ID: 19076372, 723013, 19018909, 19076374, 723042, 723020)

Valproic acid (Drug Concept ID: 40237988, 40238017)

NO = If any are presently prescribed

**DEVICES DOMAIN** **16 data elements, 2 variables**

**Derived variable: Mobility Device Used = YES if one of the following two variables is YES**:

Wheelchair independently (4012670)

Unable to propel own wheelchair (4215087)

**AND** any of the following variables are YES:

Manual wheelchair (4045112)

Power wheelchair (2616920)

NO = if “Unable to propel own wheelchair” and “Wheelchair independently” are NO

MISSING = Manual wheelchair and Power wheelchair are NO and “wheelchair independently” and “unable to propel own wheelchair” are MISSING

**Derived variable: Ambulation Device Utilized = YES if one or more of the following 6 variables are YES:**

Gait trainer (3038446)

Stander (37396481)

Swivel walker (45764219)

Walker pickup (2616486)

Walker reverse (2500010165)

Walker wheeled (2616497)

**AND** any of the following variables are NO:

None (2500010164)

Cane, quad (2500010160)

Cane, single point (2500010161)

Cane, tripod (2500010162)

Crutches axillary (45772311)

Crutches forearm (2616479)

NO = if any of the following variables are stipulated YES:

None (2500010164)

Cane, quad (2500010160)

Cane, single point (2500010161)

Cane, tripod (2500010162)

Crutches axillary (45772311)

Crutches forearm (2616479)

MISSING = if ALL the following variables are missing:

Gait trainer (3038446)

Stander (37396481)

Swivel walker (45764219)

Walker pickup (2616486)

Walker reverse (2500010165)

Walker wheeled (2616497)

**AND** any of the following variables are stipulated NO:

None (2500010164)

Cane, quad (2500010160)

Cane, single point (2500010161)

Cane, tripod (2500010162)

Crutches axillary (45772311)

Crutches forearm (2616479)

**MOBILITY DOMAIN 10 data elements, 4 variables**

**Derived Variable: Gross Motor Concerns (CONCEPT ID: 2500010797; Qualifier: 2500010797) = YES if one of the following two variables is YES:**

Unable to sit independently (4106332)

Non-ambulatory (2500010193)

**AND** if one or more of the five variables below are NO:

Ambulatory with assistance (2500010171)

Assistive devices needed (3039217)

Household ambulation (2500010186)

Tires easily (45881740)

Trips/falls frequently (2500010215)

NO = if “Unable to sit independently” AND “Non-ambulatory” are NO

MISSING = if “Unable to sit independently” is MISSING AND “Non-ambulatory” is MISSING and all other variables in this derived variable are NO or MISSING

**Variable: Ambulation, Ambulation Level (CONCEPT ID: 2500010792) = YES if any of the following two values are selected:**

Moderate assistance – 4 (2500000259)

Maximum assistance – 2 (2500000260)

Dependent – 1 (2500000261)

NO = if any of the following values are selected:

Independent-7 (2500000256)

Stand-by assistance – 6 (2500000257)

Minimal assistance – 5 (2500000258)

MISSING = if ANY of the above values are missing

**Variable: Stairs Assistance (CONCEPT ID: 2500010796) = YES if the following value is selected or the variable is MISSING:**

Dependent (4159760)

NO = if any of the following values are selected:

Complete independence (2500000143)

Standby assistance (2500000269)

Contact guard assistance (2500000270)

Minimal assistance (2500000146)

Moderate Assistance (2500000147)

Maximal Assistance (2500000145)

**Variable: Primary Mobility (CONCEPT ID:2500000268) = YES if one of the following two values is selected:**

Independent wheelchair mobility – manual (2500000360)

Independent wheelchair mobility – power (2500000361)

Dependent wheelchair mobility (2500000359)

NO = if any of the following values is selected:

Ambulation without device (2500000358)

Ambulation with device (2500000357)

Other (9177))

MISSING = if no value is stipulated for the variable

**MOTOR DOMAIN 10 data elements, 6 variables**

**Derived Variable: Drooling = YES if either of the following variables is YES:**

Nutritional Risk Factors (CONCEPT ID: 2500010706): Drooling (2500000178)

Drooling Oral Motor Function (28312333)

NO = if the above variable is NO

MISSING = if both of the variable values is MISSING

**Variable: Sitting Balance PT (4186717, (Source Value 28295811)) = YES if the following value is selected:**

Impaired

NO = if the following value is selected:

Intact

MISSING = if no value is stipulated for this variable

**Variable: Knee Flexors/Extensors** **(2500010279/2500010278) = YES if the following values are selected on the Modified Ashworth Scale:**

2

3

4

NO = if one of the following values is selected:

0

1

1.5

MISSING = if no value is stipulated in this variable

**Variable: Ankle Dorsiflexors/Plantar flexors** **(2500010262/2500010265) = YES if the following values are selected on the Modified Ashworth Scale:**

2

3

4

NO = if one of the following values is selected:

0

1

1.5

MISSING = if no value is stipulated in this variable

**Variable: Elbow flexor/extensor muscle tone (2500010267/2500010266) = YES if the following values are selected on the Modified Ashworth Scale:**

2

3

4

NO = if one of the following values is selected:

0

1

1.5

MISSING = if no value is stipulated in this variable

**Variable: General Strength, Neck (2500010813) =YES if the following value is selected:**

Limited (2500000040)

NO = if the following value is selected:

WFL (2500000041)

MISSING = if no value is stipulated in this variable
